# Liposomal Co-Delivery of Acteoside, CBD, and Naringenin: A Synergistic Strategy Against Gliomas 

**DOI:** 10.3390/pharmaceutics17081026

**Published:** 2025-08-07

**Authors:** Jagoda Szkudlarek, Ludwika Piwowarczyk, Violetta Krajka-Kuźniak, Aleksandra Majchrzak-Celińska, Szymon Tomczak, Mikołaj Baranowski, Rafał Pietrzyk, Aneta Woźniak-Braszak, Anna Jelińska

**Affiliations:** 1 Department of Pharmaceutical Chemistry, Poznan University of Medical Sciences, 3 Rokietnicka, 60-806 Poznań, Polandajelinsk@ump.edu.pl (A.J.); 2 Doctoral School, Poznan University of Medical Sciences, Bukowska 70, 60-812 Poznań, Poland; 3 Department of Pharmaceutical Biochemistry, Poznan University of Medical Sciences, 3 Rokietnicka, 60-806 Poznań, Poland; 4 Department of Functional Materials Physics, Institute of Physics, Faculty of Physics and Astronomy, Adam Mickiewicz University, Uniwersytetu Poznańskiego 2, 61-614 Poznań, Poland

**Keywords:** acteoside, cannabidiol (CBD), naringenin, cancer, glioma, glioblastoma (GBM), anticancer, drug delivery system (DDS), liposomes

## Abstract

**Background/Objectives**: Adult-type diffuse gliomas, including astrocytoma and glioblastoma multiforme (GBM), are brain tumors with a very poor prognosis. While current treatment options for glioma patients are not providing satisfactory outcomes, research indicates that natural compounds could serve as alternative treatments. However, their low bioavailability requires nanotechnology solutions, such as liposomes. **Methods**: In this study, we propose the co-encapsulation of acteoside (ACT) with other natural compounds, cannabidiol (CBD) or naringenin (NG), in a cationic liposomal nanoformulation consisting of DOTAP and POPC lipids, which were prepared using the dry lipid film method. The liposomes were characterized by their physicochemical properties, including particle size, zeta potential, and polydispersity index (PDI), with additional analyses performed using ^1^H Nuclear Magnetic Resonance (NMR). Furthermore, biological experiments were performed with U-87 MG astrocytoma and U-138 MG GBM cell lines and non-cancerous MRC-5 lung fibroblasts using the MTT assay and evaluating the expression of Bax and Bcl-xL to evaluate their potential as anticancer agents. **Conclusions**: The IC_50_ values for the nanoformulations in U-138 MG cells at 48 h were 6 µM for ACT + CBD and 5 µM for ACT + NG. ACT and CBD or NG demonstrated a potential synergistic effect against GBM in a liposomal formulation. Notably, treatment with ACT + CBD (5 µM) and ACT + NG (5 µM) liposomal formulations significantly upregulated Bax protein level in U-138 cells at both 24 and 48 h. In parallel, ACT + CBD (5 µM) also modulated Bcl-xL protein level in both U-138 MG and U-87 MG cell lines at the same time points. The obtained nanoformulations were homogeneous and stable for 21 days, evidenced by a narrow particle size distribution, a low polydispersity index (PDI) < 0.3, and a positive zeta potential.

## 1. Introduction

Diffuse gliomas are one of the most common primary malignancies of the central nervous system (CNS) [1]. Worldwide, the annual incidence of gliomas is approximately 6 per 100,000 individuals, with men being 1.6-fold more likely to be diagnosed than women. *IDH*-mutant adult-type astrocytomas are typically diagnosed in young adults, while *IDH*-wildtype GBM tumors usually occur at 50–60 years of age [2].

The standard treatment for newly diagnosed astrocytoma or GBM includes surgery to remove as much tumor as safely possible, combined with radiotherapy and chemotherapy with temozolomide (TMZ) [3]. Resection substantially improves survival, particularly when aided by fluorescence guidance; however, recurrence remains likely due to infiltrative cancer cells at the margins of resection [4]. Veviorskiy et al. reported that radiotherapy improves survival in GBM but may worsen outcomes in low-grade glioma [5]. TMZ, an oral DNA alkylating agent that crosses the blood–brain barrier (BBB), remains the standard chemotherapy, extending survival by 2.5 months [3,6]. However, radio- and chemo-resistant glioma stem cells contribute to glioma’s high mortality by surviving treatment and serving as a reservoir for glioma recurrence [5,7,8].

Moreover, the effective treatment of gliomas is challenged by several obstacles, including the highly immunosuppressive tumor microenvironment (TME) that supports glioma growth, the BBB, and extensive tumor heterogeneity [9,10]. Thus, the median survival for GBM is still around 8 months [11], with only 5.5% of patients living more than 5 years after diagnosis [3,12]. As far as the *IDH*-mutant, grade 4 astrocytomas are concerned, the median overall survival is 31.2 months, with a 5-year survival probability of only 26% [13]. Therefore, novel treatment options for diffuse gliomas are urgently needed.

In this context, a growing amount of data indicates that phytocompounds can effectively target glioma cells and improve therapy outcomes [14]. In this regard, evidence indicates that natural compounds such as acteoside (ACT), cannabidiol (CBD), and naringenin (NG) are especially promising.

Acteoside (verbascoside or orobanchin) is a water-soluble phenylethanoid glycoside found in *Verbascum sinuatum* and *Cistanche deserticola* [15,16]. ACT demonstrates various effects, such as antioxidant, neuroprotective, anti-inflammatory, and anticancer activities. The health-promoting effects of ACT are achieved by several processes, including the reduction of oxidative stress, the induction of apoptosis, the prevention of angiogenesis, the inhibition of cell invasion and metastasis, the formation of synergy with other chemical compounds, and the regulation of cell growth through the control of multiple signaling pathways such as NF-κB, MAPK, PI3K/AKT, AMPK/mTOR, and TGFβ/Smad [17,18]. In the context of glioma, ACT inhibits GBM cell proliferation, migration, and invasion while promoting apoptosis through SHP-1 activation and STAT3 phosphorylation inhibition [19]. In contrast, in another study, it decreases the viability of GBM cells and promotes autophagy by enhancing the expression of let-7g-5p and suppressing HMGA2 through inhibition of the Wnt/β-catenin signaling pathway [20].

Cannabidiol (CBD), a non-intoxicating, non-psychoactive cannabinoid derived from *Cannabis*, has the potential to enhance the effectiveness of conventional cancer therapies such as chemotherapy and radiation while protecting against neurological and organ damage [21,22]. CBD exhibits anticancer effects by interacting with the endocannabinoid system, leading to tumor cell inhibition, pain relief, and reduced chemotherapy-related side effects such as nausea and vomiting [21]. In vitro and in vivo studies have shown that CBD inhibits the growth of human glioma cells through mechanisms such as inducing apoptosis, promoting oxidative stress, blocking the lipoxygenase (LOX) pathway, modulating the endocannabinoid system, and disrupting angiogenesis related to tumor growth [23]. In an in vitro study, Solinas et al. found that CBD diminished the proliferation and invasiveness of U-87 MG and T98G cells and reduced expression of proteins related to growth, invasion, and angiogenesis, while also down-regulating ERK and Akt pro-survival pathways and decreasing hypoxia-inducible factor HIF-1α levels in U-87 MG cells [24].

**Figure 1:**
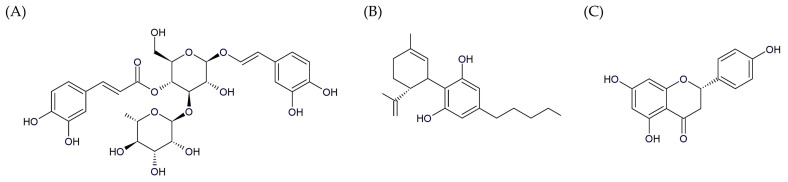
Chemical structures of compounds analyzed in this study: (**A**) acteoside (ACT); (**B**) cannabidiol (CBD); (**C**) naringenin (NG).

Naringenin (NG), a flavonoid found in a range of citrus fruits, tomatoes, bergamots, and other fruits, has been demonstrated to exert antiproliferative, pro-apoptotic, and immunomodulatory effects in glioma models [25,26,27,28]. In this context, it exhibits anti-glioma activity through modulation of the aryl hydrocarbon receptor activity and the impact on the IL-6, CCL2, and TNF-α expression [25]. The chemical structures of the above-mentioned natural compounds are shown in Figure 1.

Additionally, multi-drug therapy and synergistic drug combinations can enhance astrocytoma and GBM treatment by targeting multiple pathways, reducing resistance and toxicity while maintaining efficacy [29,30,31]. For instance, studies demonstrate that combining TMZ with perphenazine (in GBM tumorspheres) or doxorubicin (in TMZ-sensitive U-87 MG and resistant GBM43 and GBM6 cells) effectively enhances treatment efficacy [30,31]. Current literature highlights the synergistic potential of TMZ with ACT, CBD, and NG in glioma therapy. In GBM, TMZ combined with ACT triggers apoptosis and autophagy via the MAPK pathway [32]. CBD boosts TMZ’s efficacy in U-87 MG, U251, and GBM163 cells by increasing ROS, activating AMPK, raising LC3A, and inhibiting RAD51 in *MGMT*-methylated GBM, enhancing tumor sensitivity to TMZ [33]. Likewise, TMZ with NG synergistically reduces proliferation, colony formation, and migration in U-87 MG and LN229 cells more effectively than individual compounds [34].

Recent studies indicate that the incorporation of natural compounds into nanoscale drug carriers improves penetration across the BBB, allows targeted drug delivery, biocompatibility, and lowering of systemic toxicity [35]. Generally, several challenges, including low bioavailability, poor solubility, or undesirable side effects, can be effectively addressed by incorporating drugs into a drug delivery system (DDS) [36]. ACT can be delivered using nanoparticles, quantum dots, and gold nanoshells [37,38,39]. CBD has been administered via solid lipid nanoparticles, mPEG-PLGA formulations, mannose-conjugated chitosan-coated PLGA nanoparticles, and zein nanoparticles [40,41,42,43,44]. NG has been incorporated into delivery systems such as PLGA nanoparticles, nanosuspensions, solid lipid nanoparticles, and lipid nanocarriers [45,46,47,48]. Among the various DDS, liposomes, defined as spherical bilayer vesicles, are a widely utilized component of DDS due to their straightforward synthesis, controllable composition and size, simple surface modifications, biocompatibility, and non-toxicity [36]. Notably, liposomes can encapsulate both hydrophilic (e.g., ACT) and hydrophobic (e.g., CBD, NG) compounds, thereby enhancing the stability, bioavailability, and therapeutic effectiveness of these plant-derived substances [36,49]. Additionally, to improve the ability of liposomes to cross the BBB, various surface modifications, such as using long-circulating, specifically targeted, or cationic liposomes, can be employed instead of conventional formulations. Cationic liposomes can enhance pharmaceutical agent transport across the BBB by maximizing the retention of liposome-endothelial tissue interactions. The presence of cationic lipids and the size of the particles play a crucial role in achieving effective drug loading and delivery to the brain [50]. According to Joshi et al. the maximum cationic charge is not essential for efficient brain tumor uptake; a moderate cationic lipid molar fraction was found to be optimal [51].

In our previous study, we demonstrated the anti-glioma potential of curcumin, bisdemethoxycurcumin, ACT, and orientin, with liposomal ACT showing the most potent cytotoxicity against T98G cells (IC_50_ = 2.9 ± 0.9 µM, 24 h) [52]. This study continues the research on encapsulating natural compounds in liposomes, focusing on ACT and its co-encapsulation with CBD and NG in liposomal carriers for drug delivery. Thus, the aim of this study was to obtain, characterize, and test novel liposomal nanoformulations of ACT, CBD, and NG in the context of gliomas. Here, we provide evidence that liposomal ACT, CBD, and NG can easily be obtained through the dry-film method and that they exert interesting anti-glioma potential, worth exploring in further in vivo studies.

## 2. Materials and Methods

### 2.1. Chemical Compounds and Reagents

Acteoside (ACT), (2R,3R,4R,5R,6R)-6-[2-(3,4-dihydroxyphenyl)ethoxy]-5-hydroxy-2-(hydroxymethyl)-4-{[(2S,3R,4R,5R,6S)-3,4,5-trihydroxy-6-methyloxan-2-yl]oxy}oxan-3-yl-(2E)-3-(3,4-dihydroxymethyl)prop-2-enoate, was purchased from Tokyo Chemical Industry Co., Ltd., Tokyo, Japan.

Cannabidiol (CBD), 2-[(1R,6R)-3-methyl-6-prop-1-en-2-ylcyclohex-2-en-1-yl]-5-pentylbenzene-1,3-diol, was purchased from Medcolcanna Organics Inc., Distrito Especial, Bogotá, Colombia.

Naringenin (NG), (2S)-5,7-dihydroxy-2-(4-hydroxyphenyl)-2,3-dihydrochromen-4-one, was obtained from Sigma Aldrich, Saint Louis, MO, USA.

1-Palmitoyl-2-oleoyl-glycero-3-phosphocholine (POPC) and 1,2-dioleoyl-3-trimethylammonium-propane (DOTAP) were obtained from Avanti Polar Lipids (Birmingham, AL, USA). Chloroform, acetonitrile, and methanol were obtained from Sigma-Aldrich (St. Louis, MO, USA) to prepare stock solutions for liposome preparation.

HPLC-grade acetonitrile and water, were procured from Avantor Performance Materials (Gliwice, Poland), and 85% phosphoric acid was purchased from Merck KGaA (Darmstadt, Germany).

Reagents used for in vitro experiments, such as phosphate-buffered saline (PBS) and 3-(4,5-dimethylthiazol-2-yl)-2,5-diphenyltetrazolium bromide (MTT), were obtained from Sigma Aldrich.

### 2.2. Liposome Preparation

Briefly, the lipids and chemical compounds were dissolved in chloroform (CBD), acetonitrile (NG), and methanol (ACT) in the specified molar ratio, the solvent was evaporated (25 min.), and the resulting film dried in a vacuum (10 min.). After that, it was rehydrated using PBS, vortexed (1 min. 3000 rpm), and sonicated in an ultrasonic bath (2 min/180 W) until the uniform suspension was achieved. Next, the particle size was unified using an Avanti^®^ Polar Lipids Mini Extruder (Merck KGaA, Darmstadt, Germany) with 100 nm polycarbonate membranes, according to the manufacturer’s instructions. The average diameter, PDI, and zeta potential of all liposomes were measured at four time points: T0 (day 0, immediately after preparation), T7 (day 7), T14 (day 14), and T21 (day 21)—to evaluate their stability throughout the experiment.

Liposomes were obtained using a method outlined in the literature [53] using a mixture of DOTAP and POPC in a 2:8 ratio, along with either ACT or a combination of ACT with CBD or NG, with the following molar ratios: 0.1:2:8 (ACT:DOTAP:POPC), 0.08:0.02:2:8 (ACT:NG:DOTAP:POPC), and 0.05:0.05:2:8 (ACT:CBD:DOTAP:POPC). The final concentrations of the components were as follows: 62.5 µg/mL ACT, 1397.1 µg/mL DOTAP, 6080.6 µg/mL POPC, and for the compound mixtures, 31.2 + 15.7 µg/mL ACT + CBD and 50.0 + 5.4 µg/mL ACT + NG.

### 2.3. Liposome Size, PDI, and Zeta Potential Measurements

Dynamic light scattering (DLS) was employed to evaluate particle size and PDI, while zeta potential was determined through electrophoretic light scattering (ELS) using the Zetasizer Nano ZS (Malvern Instruments, Malvern, UK). All analyses were repeated three times. To obtain the optimal particle concentration, 10 µL of liposome suspension was mixed with 10 mL of water. Measurements were taken using U-shaped cuvettes equipped with a gold electrode.

### 2.4. HPLC Assay for Encapsulation Efficiency

The Agilent Infinity II 1260 (Altium International, Warsaw, Poland) instrument was used to determine the encapsulation efficiency. The HPLC system consisted of a 1260 Infinity III Diode Array Detector WR (Agilent, Santa Clara, CA, USA), an autosampler, a binary pump, and a column oven. The flow rate was 0.75 mL/min, and the gradient elution used a mobile phase consisting of 0.1% (*V*/*V*) phosphoric acid solution (phase A) and acetonitrile (phase B) at an initial ratio of 88%/12%. After 8 min of isocratic elution, a gradient was started up to 15 min with a decrease of phase A up to 20% and then 10 10-min isocratic flows. From 25 to 30 min gradient elution returned to the initial ratio (88/12%), and 5 min for isocratic elution, prepared the column for another injection. The LIchrospher^®^ 100 RP-18 E (125 × 4 mm; 5 µm; Merck KGaA Darmstadt, Germany) column contained a stationary phase of octadecylsilane-modified endcapped silica gel for chromatography and was stored at 25 ± 1 °C. All three analyzed compounds were determined in one run (analysis duration 35 min), but detection was performed at different wavelengths: for CBD—220 nm, for NG—290 nm, and for ACT—320 nm. An appropriately prepared sample containing loaded liposomes (100 µL) was mixed with 0.5 mL of methanol to release the active substance and then subjected to HPLC analysis, in which 10 µL of the sample was injected into the column. Before determining the encapsulation efficiency, the method was validated so that the concentrations of active substances in liposomes were in the mid-linearity range. The method was validated for selectivity, precision, and linearity according to the ICH Q2(R2) guidelines “Validation of analytical methods—scientific guideline” [54].

The efficiency of encapsulation (EE) for ACT, ACT + CBD, and ACT + NG was assessed based on a standard calculation formula [55]:

*EE* = *C_en_*/*C_in_* × 100%

where *C_en_* is the actual amount of the substance measured by means of HPLC in the liposomes after their disruption and *C_in_* is the initial amount of the substance used for the preparation of the liposomes.

### 2.5. ^1^H Nuclear Magnetic Resonance

#### 2.5.1. NMR Relaxation Measurements

Nuclear Magnetic Resonance (NMR) is a powerful and versatile technique for studying liposomal systems [56,57,58,59]. Its unique ability to probe molecular dynamics, structural organization, and intermolecular interactions at the atomic scale makes it especially valuable. Through measurements of relaxation times (T_1_, T_2_) and diffusion coefficients, NMR provides direct insight into molecular mobility, phase transitions, and compartmentalization within these self-assembled structures. The NMR technique is particularly sensitive to changes in the local environment, allowing for the detection of even subtle differences in aggregation state, particle size, and molecular packing. Additionally, NMR can distinguish between different physical states, such as rigid bilayers versus fluid cores, based on characteristic relaxation behaviors. This non-invasive and highly quantitative approach makes NMR an indispensable tool for elucidating the dynamic and structural properties of complex colloidal systems such as liposomes, which are otherwise challenging to characterize using conventional imaging or scattering techniques.

Molecular dynamics within these structures were examined by determining relaxation parameters such as the spin-lattice T_1_ and spin-spin T_2_ relaxation times measured in the laboratory frame [60].

The spin-lattice relaxation time T_1_ characterizes the efficiency of energy transfer from the nuclear spin system to surrounding molecules. In contrast, the spin-spin relaxation time T_2_ relates to energy-conserving interactions between nuclei, leading to the gradual dephasing of spinning dipoles and the exponential decay of magnetization within the transverse (xy) plane.

The spin-spin relaxation time T_2_ is directly proportional to the linewidth at half-height (∆ν_1/2_) of the NMR signal, following the relationship (T_2_~1/∆ν_1/2_). In liquid systems, isotropic motions, such as molecular tumbling or lateral diffusion, contribute to the averaging of NMR spectra, resulting in relatively long T_2_ values, typically ranging from tens of milliseconds to several seconds. For systems such as liposomes, typical T_1_ values are around 100 ms to 500 ms. If the systems being studied are very stiff, T_1_ can be as short as less than 100 ms. If systems are very fluid (e.g., high temperature, liquid lipids), T_1_ can increase to 1–2 s [61,62]. In liposomal systems, increased intermolecular interactions, including van der Waals forces, hydrogen bonding, and electrostatic effects, restrict molecular dynamics by limiting the rotational and translational mobility of molecules within the structure [63]. This restriction enhances dipolar interactions and reduces motional averaging, resulting in increased local magnetic field inhomogeneities. Consequently, NMR spectral lines become broader due to faster dephasing of transverse magnetization. This effect manifests as a significant reduction in T_2_ relaxation times, reflecting the transition from predominantly motional narrowing regimes to those dominated by spin–spin interactions and static disorder. Such behavior is characteristic of condensed or semi-solid systems, where reduced molecular mobility directly correlates with shorter transverse relaxation times and broader resonance lines.

Proton NMR experiments were conducted using a pulsed spectrometer operating at 30.2 MHz. The spin-lattice relaxation time T_1_ in the laboratory frame was measured using a conventional saturation pulse sequence $\left(n\times 90_{x}^{0}-\tau -90_{x}^{0}\right).$ The values of the relaxation times T_1_ were determined by fitting the following equation to the experimental data:

(1) $$M\left(t\right)=M_{0}\left(1-exp\left(-\frac{t}{T_{1}}\right)\right)$$

where $M_{0}$ represents the equilibrium magnetization. The uncertainty in the measurements was estimated to be within a few percent [64].

#### 2.5.2. NMR Off-Resonance

The NMR off-resonance method is an effective approach for studying rotational correlation times in liquids.

This method is particularly effective for studying molecular dynamics, including intermediate motions with correlation times ranging from approximately 1 to 2 nanoseconds, which are challenging to assess using conventional relaxation techniques.

In this technique, an RF field is applied at a frequency ω, different from the resonance frequency from the nuclear spin, creating a rotating frame of reference. This approach allows for the selective observation of specific relaxation processes, providing insights into molecular reorientation times without interference from other relaxation mechanisms. The off-resonance rotating-frame spin-lattice relaxation time $T_{1\rho }^{off}$ is then measured and analyzed to extract information about molecular dynamics [65,66,67].

Moreover, this method enables the determination of the correlation times at one temperature with no need of temperature changes, which would irreversibly alter the structure of the biological materials. An important scientific aspect of the off-resonance measurements was the determination of molecular motion parameters from the measurements of the equilibrium value of magnetization $M_{\rho }$ in different effective fields $B_{ef}$ in the rotating frame. The off-resonance NMR technique and its application have been described in detail in some articles [64,68,69].

The effective magnetic field $B_{ef}$ in the rotating frame can be expressed as a sum of the following components:

(2) $$B_{ef}=\;B_{1}\hat{i}+\Delta B\hat{k}$$

where *B*_1_ denotes the amplitude of the oscillating radiofrequency (RF) field component that is perpendicular to the static magnetic field $B_{0}$, which is oriented along the *z*-axis. The ∆B, component, aligned parallel to the magnetic field $B_{0}$, has an amplitude proportional to the difference between the resonance angular frequency $\omega_{o}$ and the angular frequency ω of field $B_{1}.$ This component is described by the following expression:

(3) $$∆B=\frac{\omega_{0}-\omega }{\gamma }=\frac{∆f}{2\pi \gamma }$$

The effective field $B_{ef}$ is inclined at an angle *θ* to the static magnetic field $B_{0}$:

(4) $$\theta =arctan\left(\frac{B_{1}}{∆B}\right).$$

The spin system, placed in an external static magnetic field $B_{0}$, is characterized by an equilibrium magnetization $M_{0}$. In the NMR off-resonance experiment conducted in the rotating frame, applying the oscillating RF field $B_{1}$ at the off-resonance frequency $f=\frac{\omega }{2\pi }$, causes the magnetization vector to be displaced from its equilibrium position. It then begins to precess around the effective field $B_{ef}$. The precessing magnetization vector $M_{0}$ can be resolved into two components relative to $B_{ef}$: a parallel component $M_{II}(t)$ and a perpendicular component $M_{⊥}\left(t\right).$ The perpendicular component of magnetization $M_{⊥}(t)$ decays rapidly with a time constant $T_{2\rho }^{off}$ whereas the parallel component $M_{II}(t)$ remains locked along the effective field $B_{ef}$ and relaxes towards its equilibrium value $M_{\rho }$ with the time constant $T_{1\rho }^{off}$.

To investigate the molecular dynamics of liposomal systems, the measurements of the equilibrium magnetization $M_{\rho }$ in the effective magnetic field $B_{ef}$ as a function of distance from the resonance frequency Δf were carried out using a homemade pulse spectrometer operating at 30.2 MHz.

The experiment was performed for: $\omega_{e}\tau_{c}\ll 1$ and the $B_{1}$ field of 5Gs, which was constant for each preset value of the frequency Δf, at the constant temperature T = 303 K.

**Figure 2:**
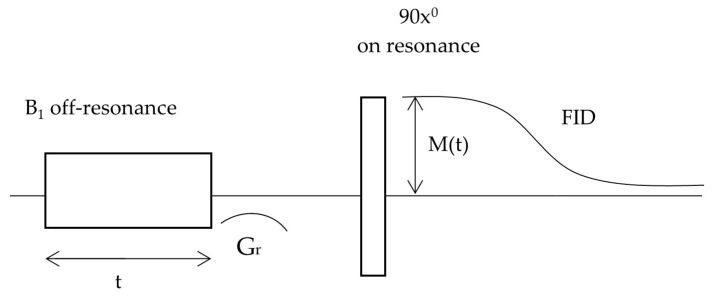
The measurement sequence used in the off-resonance experiment.

The pulse sequence employed in this experiment is illustrated in Figure 2. The initial radiofrequency (RF) pulse was applied off-resonance, creating the effective magnetic field $B_{ef}.$ After about 1.4 ms, a 90° on-resonance pulse along the *x*-axis was applied. Between the off-resonance and on-resonance pulses, a magnetic field gradient Gr was switched on to suppress the transverse components of the magnetization.

Following the second RF pulse, a free induction decay (FID) signal was observed, whose amplitude M(t) depended on the duration t of the initial off-resonance RF pulse. The equilibrium magnetization $M_{\rho }$ was obtained when the duration *t* equaled approximately 5 times the spin-lattice relaxation time T_1_.

The ratio of intensities $M_{\rho }/M_{0}$ depends on the $B_{1}$ field and can be expressed as follows [68]:

(5) $$\frac{M_{\rho }}{M_{0}}=\frac{∆f^{2}}{∆f^{2}+K{\left(\frac{\gamma }{2\pi }\right)}^{2}\cdot B_{1}^{2}}$$

The constant *K*, which contains information about the correlation time $\tau_{c}$, is given by the following expression:

(6) $$K=\frac{10+37\omega^{2}\tau_{C}^{2}+12\omega^{4}\tau_{C}^{4}}{10+16\omega^{2}\tau_{C}^{2}}$$

The correlation time $\tau_{c}$ was calculated from Equation (6).

### 2.6. Biological Activity Assessment

#### 2.6.1. Cell Culture and Viability Assay

The human glioma cell lines U-87 MG and U-138 MG were purchased from the American Type Culture Collection (ATCC, Manassas, VA, USA), while the normal human lung fibroblast cell line MRC-5 was obtained from the European Collection of Authenticated Cell Cultures (ECACC), distributed by Sigma-Aldrich (St. Louis, MO, USA). Glioma cell lines were cultured in Eagle’s Minimum Essential Medium (EMEM), while normal cells in Dulbecco’s Modified Eagle’s Medium (DMEM) supplemented with 10% fetal bovine serum (FBS; EURx, Gdańsk, Poland) and 1% penicillin-streptomycin solution (Sigma-Aldrich), under standard cell culture conditions (37 °C, 5% CO_2_, and humidified atmosphere).

Cell viability was assessed using the MTT [3-(4,5-dimethylthiazol-2-yl)-2,5-diphenyltetrazolium bromide] assay according to a standardized procedure. Briefly, U-87 MG, U-138 MG, and MRC-5 cells were seeded into 96-well plates at a density of 1 × 10^4^ cells per well and allowed to adhere for 24 h in complete medium. Subsequently, cells were exposed to the test compounds and incubated for an additional 24 h. After treatment, cells were rinsed twice with phosphate-buffered saline (PBS) and incubated for 4 h with EMEM containing 0.5 mg/mL MTT. The resulting formazan crystals were solubilized in acidified isopropanol, and the absorbance was recorded at 570 nm with a reference wavelength of 690 nm using an Infinite M200 microplate reader (Tecan, Grödig, Austria). All experiments were conducted in biological triplicates. Cell viability was calculated and expressed as the percentage of viable cells relative to the untreated control group. IC_50_ values are presented in the results.

#### 2.6.2. Total Protein Lysate Preparation

The glioma cell lines U-87 MG and U-138 MG were treated with ACT, the combination ACT + CBD, and ACT + NG, and the control (DOTAP:POPC) nanoformulation for 24 h and 48 h.

Total cellular protein was extracted using radioimmunoprecipitation assay (RIPA) buffer supplemented with a protease inhibitor cocktail (Sigma-Aldrich, St. Louis, MO, USA), according to the manufacturer’s recommendations. Protein concentrations were quantified using a bicinchoninic acid (BCA) assay or an equivalent standard colorimetric method. Lysates were aliquoted and stored at −80 °C until further analysis.

#### 2.6.3. Western Blot Assay

A western blot analysis was conducted to determine Bax and Bcl-xL protein levels.

Protein lysates were resolved by SDS-PAGE on 10% or 12% polyacrylamide gels and subsequently transferred onto nitrocellulose membranes using standard wet transfer conditions. Membranes were blocked for 1 h at room temperature in 10% (*w*/*v*) non-fat dry milk prepared in Tris-buffered saline containing 0.1% Tween-20 (TBS-T) and then incubated overnight at 4 °C with primary antibodies targeting Bax, Bcl-xL, and β-Actin. β-Actin was used as an internal loading control to normalize protein levels. Following primary antibody incubation, membranes were washed and incubated with species-specific horseradish peroxidase (HRP)-conjugated secondary antibodies (anti-goat IgG or anti-rabbit IgG, as appropriate). Immunoreactive protein bands were detected using the Clarity Western ECL Substrate (Bio-Rad, Hercules, CA, USA) and visualized with the ChemiDoc Imaging System (Bio-Rad Laboratories, Hercules, CA, USA).

Densitometric analysis was performed, and values were expressed as relative quantification (RQ) per mg of protein, presented as a percentage relative to the control.

## 3. Results and Discussion

### 3.1. Liposome Size, PDI, EE, and Zeta Potential Measurements

A heterogeneous population of multilamellar vesicles (MLVs) was obtained using the thin-film hydration technique. The approach commences with forming a dry lipid layer, which is then hydrated using an aqueous solution. This process is accompanied by intense shaking and treatment in an ultrasonic bath. Subsequently, the extrusion technique was employed to produce a homogeneous population of liposomes, either small (SUVs) or large unilamellar vesicles (LUVs).

The liposomes were characterized by particle size, PDI, and zeta potential to assess the uniformity and stability of the obtained nanoformulation. The measurements were performed in triplicate directly before conducting the biological assays. Consistent and reproducible results are paramount to ensure the reliability of subsequent in vitro analyses.

**Figure 3:**
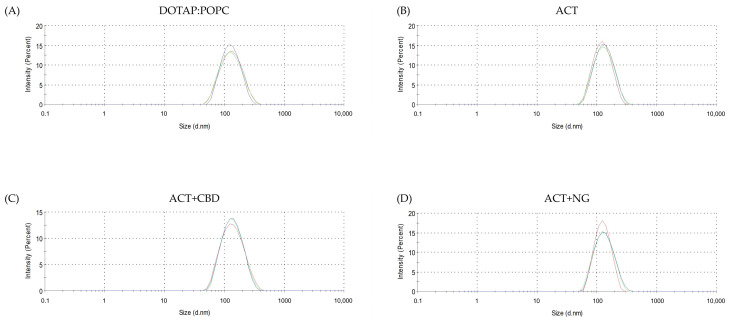
Liposome size distribution measured by dynamic light scattering (DLS) at T0: (**A**) Control DOTAP:POPC, (**B**) ACT, (**C**) ACT and CBD mixture, and (**D**) ACT and NG mixture.

The results in T0 for control liposomes (DOPAT:POPC) and liposomes with ACT and a mixture of ACT and CBD or NG liposomes are demonstrated in Figure 3.

**Figure 4:**
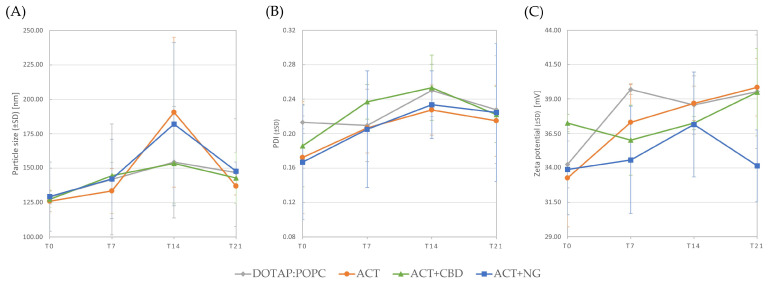
Particle size (**A**), PDI (**B**), and zeta potential (**C**) of liposomes with ACT and the mixture of ACT and CBD or NG at four measurement times (T0, T7, T14, and T21).

Table 1 and Figure 4 present the particle size, PDI, and zeta potential of the control liposomes, liposomes containing ACT, and liposomes co-encapsulating ACT with CBD or NG, measured at four time points: T0, T7, T14, and T21.

Suitability of the nanocarrier formulations for a given drug delivery route was assessed based on average size, PDI, and stability, as these factors play a critical role in ensuring their clinical applicability [70].

The tested compounds exhibit low bioavailability. In particular, the oral bioavailability of ACT was determined to be just 0.12% following the intravenous and oral administration of doses of 3 mg/kg and 100 mg/kg, respectively [71]. Additionally, its susceptibility to hydrolysis hinders the clinical use of ACT, but liposomal encapsulation enhances its stability by shielding it from degradation. Isacchi et al. demonstrated in rats that liposomal ACT (100 mg/kg, i.p.) produced a longer-lasting antihyperalgesic effect than the same dose in saline [72].

Sitovs et al. pointed out that free CBD exhibits chemical instability and low bioavailability, amounting to approximately 13–19% [73]. The authors also indicate that these challenges can be overcome by using a lipid-based delivery system for CBD. Zgair et al. demonstrated that CBD concentrations in plasma and brain in animals are dose-dependent, and bioavailability increases when various lipid-based formulations are used [74,75].

NG has low water solubility and limited oral bioavailability (around 5.8%) due to its predominantly hydrophobic ring structure [76]. Wang et al. found that liposomal NG enhanced its solubility and oral bioavailability, with a 13.44-fold increase in AUC compared to the free drug. The formulation improved release and absorption compared to free NG and showed high liver accumulation [77]. Notably, NG can cross the BBB and facilitate drug delivery to the CNS when combined with appropriate nanocarriers, effectively addressing the challenges of glioma through NG-loaded nanoparticles [78].

In the present study, throughout the 21-day experimental period, the liposomes exhibited an average particle size below 200 nm and a narrow size distribution at all measurement points (T0, T7, T14, and T21). This uniformity in size confirms both the homogeneity of the formulation and its stability over time. Additionally, for clinical injectable applications, liposome diameters should remain below 200 nm [79].

The PDI reflects the quality of a sample based on its particle size distribution, ranging from 0.0 (uniform size) to 1.0 (highly diverse sizes). A PDI value of 0.3 or below is acceptable in lipid-based drug delivery systems (LDDS) such as liposomes, indicating a homogeneous population of vesicles [70]. In the present study, throughout the entire experimental period, the PDI values ranged between 0.17 and 0.25, emphasizing the homogeneity of the obtained nanoparticles.

Zeta potential is commonly employed to estimate the surface charge of nanoparticles, indicating their character as cationic, anionic, or neutral in nature. The zeta potential directly correlates with the molar percentage of ionic lipids incorporated into liposomes, ensuring nanoparticle suspensions’ stability when values are below −30 mV or above +30 mV [80,81]. In the present study, the zeta potential values of the entire experimental period ranged between +33.3 mV and +39.9 mV, emphasizing the stability of the obtained nanoparticles.

The encapsulation efficiency for the using concentrations was 84.83 ± 1.68% for ACT, 85.23 ± 0.81% for ACT, and 100.26 ± 0.65% for CBD from the mixture (ACT + CBD), and 85.70 ± 0.82% for ACT and 99.19 ± 0.33% for NG from the mixture (ACT + NG).

### 3.2. ^1^H Nuclear Magnetic Resonance Results

#### 3.2.1. Analysis of NMR Relaxation Measurements 

**Figure 5:**
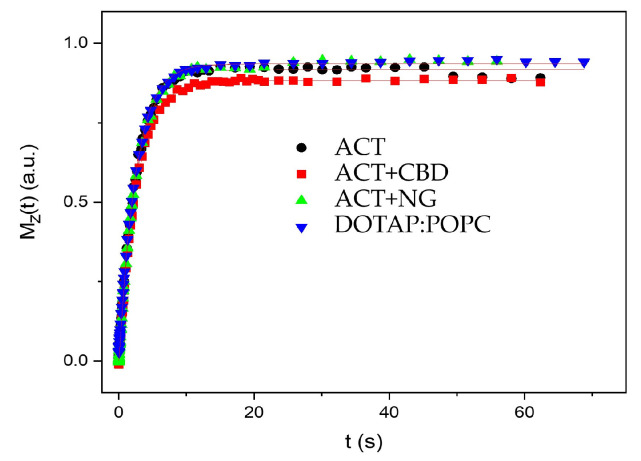
The recovery of the magnetization as a function of time *t* for all liposomal systems.

**Figure 6:**
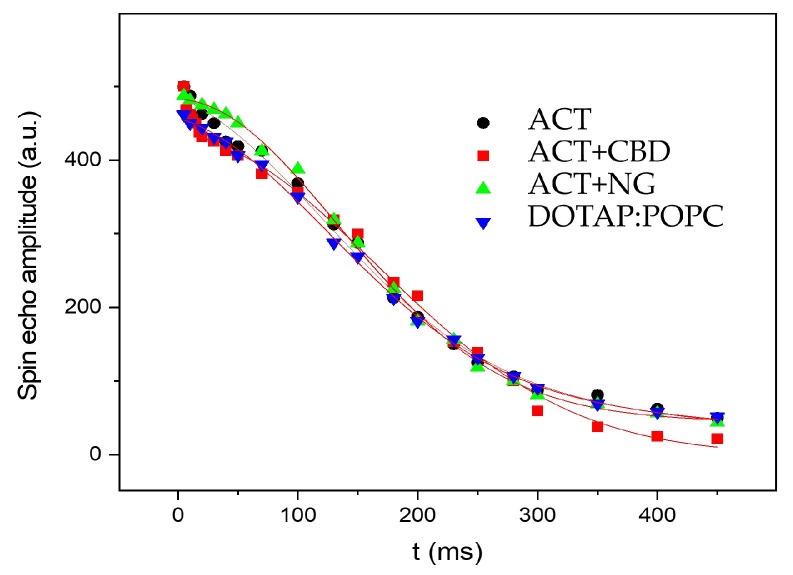
The magnetization decay observed in the experiment measuring the spin-spin relaxation time T_2_ for all liposomal systems.

In Figure 5, the recovery of magnetization as a function of time *t* is shown for various liposomal systems. The spin-spin relaxation time T_2_ in the laboratory frame was measured using the standard spin-echo sequence $\left(90_{x}^{0}-\tau -180_{x}^{0}\right)$. Figure 6 displays the corresponding magnetization decay observed in these experiments. To extract the T_2_ values, the decay curves of the magnetization were fitted using a combination of Gaussian and Lorentzian functions:

(7) $$M\left(t\right)=M_{0G}exp\left(-{\left(\frac{t}{T_{2G}}\right)}^{2}\right)+M_{0L}exp\left(-\frac{t}{T_{2L}}\right)$$

This model was employed because it captures the coexistence of two distinct relaxation mechanisms within the sample. The Gaussian component describes dephasing processes that are dominated by static field inhomogeneities, restricted molecular motion, or dipolar broadening in relatively rigid environments. Such mechanisms lead to a characteristic non-exponential decay that is symmetric and faster at early times. In contrast, the Lorentzian component accounts for relaxation processes governed by dynamic molecular motions and homogeneous broadening, where spin-spin interactions decay exponentially over time. By fitting the magnetization decay with both Gaussian and Lorentzian terms, it becomes possible to distinguish between contributions from more ordered (rigid) and more disordered (mobile) regions of the material, providing a more complete description of the underlying molecular dynamics.

For a better understanding of the molecular dynamics of such complex systems, the authors decided to use the technique of off-resonance as an excellent complement to standard NMR methods. The off-resonance NMR technique provides valuable insight into the molecular dynamics of liposomal systems [82] by selectively perturbing the magnetization away from resonance conditions. By applying an RF field at a frequency offset relative to the Larmor frequency, it becomes possible to probe slow molecular motions and weak interactions that are otherwise masked under standard resonance conditions. Off-resonance experiments are particularly sensitive to rotational correlation times and local environmental fluctuations, enabling the differentiation between rigid and mobile domains within self-assembled structures. This approach is especially useful for studying heterogeneous systems such as liposomes, where distinct dynamic populations coexist and influence the overall relaxation behavior. It should be mentioned that this method is particularly useful for determining the molecular dynamics of biological systems due to the possibility of determining molecular dynamics and correlation times at constant temperature.

The line shape in NMR spectra can be influenced by contributions from both solid and liquid phase components. Consequently, accurately identifying and distinguishing these contributions is crucial for proper spectral interpretation.

The determined relaxation times T_1_ and T_2_ are listed in Table 2.

The NMR relaxation analysis reveals notable differences in molecular dynamics across the samples. T_1_ values are relatively consistent among all materials (2.5–2.7 s), indicating similar spin-lattice relaxation mechanisms, most likely dominated by dipole–dipole interactions in a comparable molecular environment. In the G-phase, the highest relative amplitude (*M*_0*G*_ = 86%) is observed in sample ACT + NG, followed by ACT + CBD (77%), suggesting more ordered molecular domains in these systems. ACT + CBD also exhibits the longest transverse relaxation time for this phase (T_2G_ = 232 ms), indicating slower dephasing and enhanced structural rigidity, likely related to CBD’s known capacity to modulate cellular environments and neurochemical pathways [54], which may involve reduced molecular mobility due to altered intermolecular interactions. In contrast, ACT, DOTAP:POPC, and ACT + NG show comparable T_2G_ values (184–188 ms), implying moderately faster relaxation, possibly due to greater molecular motion or weaker spatial confinement. The L-phase behavior varies significantly: ACT+CBD presents the smallest fraction (*M*_0*L*_ = 23%) with a very short T_2L_ of 7.6 ms, suggesting that spins lose phase coherence rapidly and NMR signals become broad. It was suggested that molecular motion was restricted and the system became more rigid, dense, or heterogeneous due to phase separation or the presence of small mobile CBD-rich regions. Restricted molecular motion and increased heterogeneity could hypothetically arise from microphase separation or formation of CBD-rich regions, mechanisms that align with CBD’s documented interactions with membrane components and influence on cellular microenvironments [83]. Conversely, ACT + NG has a low L-phase content (14%), but the time T_2L_ is equal to 118 ms, implying also restricted molecular mobility in this minor phase, possibly due to NG-induced compartmentalization or gel-like microenvironments. ACT and DOTAP:POPC show the largest L-phase fractions (42% and 38%, respectively) and intermediate T_2L_ values (300–352 ms), associated with greater molecular dynamics and rotational and translational movements, which indicates that the liposomal system is more homogeneous.

Since the spin-spin relaxation time T_2_ is inversely proportional to the linewidth, longer T_2_ values suggest isotropic motion, such as molecular tumbling or lateral diffusion, which leads to spectral averaging in NMR. In pure liposomes, the presence of a mobile fraction is reflected in extended T_2_ relaxation times. In contrast, the significantly shorter T_2_ values observed in liposomal systems containing embedded substances indicate line broadening, likely caused by restricted anisotropic motion or liposome aggregation.

#### 3.2.2. NMR Off-Resonance Results

According to Equation (6), the relaxation enhancement factor *K* is described as a function of the angular frequency *ω* and the molecular correlation time $\tau_{c}$. The angular frequency *ω* corresponds to the offset precession frequency of the magnetization vector in the effective magnetic field, expressed in radians per second. The correlation time $\tau_{c}$ characterizes the average time over which molecular motions remain correlated; short $\tau_{c}$ values indicate rapid molecular dynamics, while longer $\tau_{c}$ values reflect slower, more restricted motions. The constants in the numerator and denominator (10, 37, 12, and 16) arise from the theoretical treatment of dipolar relaxation mechanisms and account for different contributions of motion at various frequencies. As factor *K* increases beyond 1, it indicates that molecular motions are slowing down relative to the applied frequency, leading to enhanced spin relaxation. Thus, by analyzing *K* as a function of frequency, it becomes possible to extract detailed information about molecular mobility and dynamic heterogeneity within the system, especially in complex assemblies such as liposomes.

**Figure 7:**
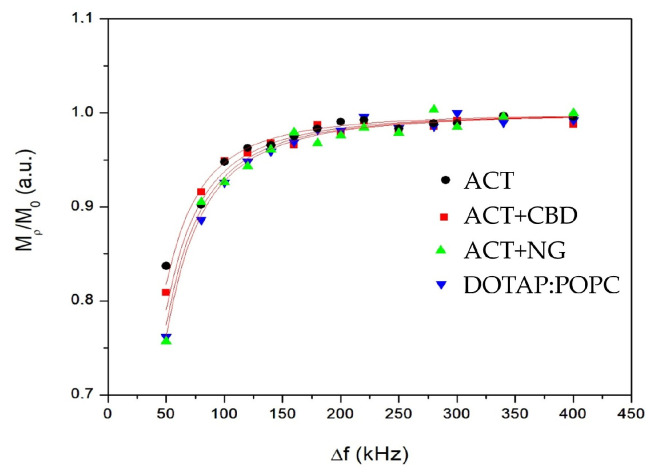
The dependency of proton ^1^H intensity ratio *M_ρ_*/*M*_0_ as a function of frequency offset from resonance Δ*f* for all investigated liposomes.

Obtained off-resonance results are presented in Figure 7.

The obtained values of the correlation times are collected in Table 3.

The NMR off-resonance measurements provide insights into the molecular dynamics of the investigated materials by characterizing the effective local motions through the relaxation enhancement factor *K* and the correlation time $\tau_{c}$. The *K* values increase systematically across the materials, starting from 1.22 ± 0.17 for ACT, through 1.46 ± 0.27 for ACT + CBD, to 1.72 ± 0.21 for ACT + NG. DOTAP:POPC also shows an elevated *K* value of 1.61 ± 0.22, close to that of ACT + NG. A higher *K* value indicates greater relaxation enhancement, which reflects more significant local molecular fluctuations interacting with the off-resonance radiofrequency field. The correlation times $\left(\tau_{c}\right)$ show a parallel trend, increasing from 1.8 ns in ACT to 3.6 ns in ACT + NG. DOTAP:POPC exhibits an intermediate $\tau_{c}$ of 3.2 ns, whereas ACT + CBD shows 2.8 ns. This trend suggests that molecular motions in ACT are the fastest, with the shortest $\tau_{c}$, while ACT+NG displays the slowest dynamics, characterized by the longest $\tau_{c}$. The observed correlation between increasing *K* values and longer $\tau_{c}$ times implies that the modifications (CBD or NG addition) introduce structural changes that slow down molecular motions. In particular, the highest *K* and $\tau_{c}$ in ACT + NG suggest that NG incorporation leads to the most significant restriction of molecular dynamics, possibly due to enhanced intermolecular interactions or increased local viscosity. Similarly, the increase in both *K* and $\tau_{c}$ for ACT + CBD compared to ACT indicates that CBD also imparts additional molecular ordering or confinement, although to a lesser extent than NG. Overall, the off-resonance data demonstrate that additives such as CBD and NG alter the molecular dynamics within the materials, promoting slower motions and stronger interaction networks, while ACT alone remains the most dynamically flexible system. An increase in the $\tau_{c}$ may also be associated with an increase in liposome size, as confirmed by DLS data.

### 3.3. Biological Activity

#### 3.3.1. Impact of Nanoformulations on Cell Viability

**Figure 8:**
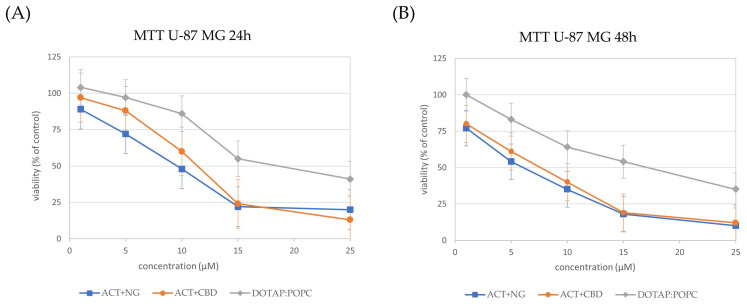
Cell viability in the U-87 MG cells incubated for 24 h (panel (**A**)) and 48 h (panel (**B**)) with nanoformulations. Cell viability was normalized to the control group treated with the empty liposomes (100% viability). Data are expressed as the mean ± standard error of the mean (SEM) based on three biologically independent replicates.

**Figure 9:**
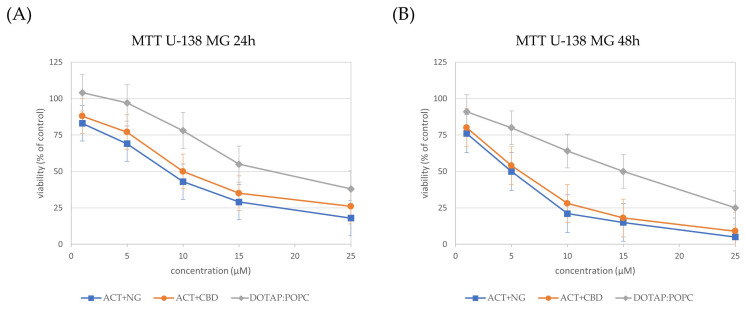
Cell viability in the U-138MG cells incubated for 24 h (panel (**A**)) and 48 h (panel (**B**)) with nanoformulations. Cell viability was normalized to the control group treated with the empty liposomes (100% viability). Data are expressed as the mean ± standard error of the mean (SEM) based on three biologically independent replicates.

**Figure 10:**
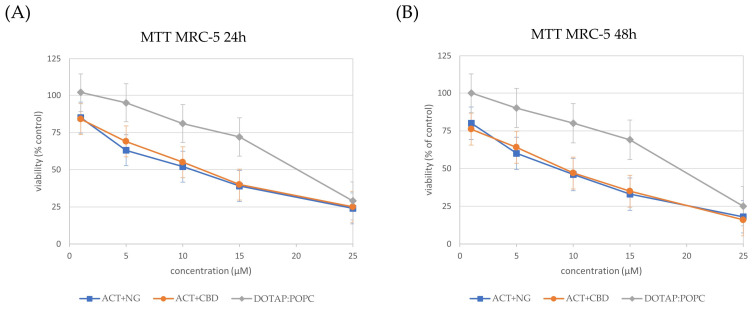
Cell viability in the MRC-5 cells incubated for 24 h (panel (**A**)) and 48 h (panel (**B**)) with nanoformulations. Cell viability was normalized to the control group treated with the empty liposomes (100% viability). Data are expressed as the mean ± standard error of the mean (SEM) based on three biologically independent replicates.

The influence of different nanoformulations on the viability of U-87 MG (Figure 8), U-138 MG (Figure 9), and MRC-5 (Figure 10) cells was assessed by MTT analysis and the IC_50_ values (Table 4) were determined after 24 and 48 h of treatment.

In both glioma cell lines (U-87 MG and U-138 MG), the ACT + NG nanoformulation demonstrated the strongest cytotoxic effect, as evidenced by the lowest IC_50_ values. Specifically, in U-87 MG cells, ACT + NG nanoformulation reduced the IC_50_ from 9 µM at 24 h to 6 µM at 48 h, while in U-138 MG cells, IC_50_ values decreased from 8 µM to 5 µM over the same period of time. The ACT + CBD combination also decreased cell viability but was slightly less potent, with IC_50_ values of 11 µM and 7 µM in U-87 MG cells and 10 µM and 6 µM in U-138 MG cells at 24 and 48 h, respectively.

The control (DOTAP:POPC) formulation displayed significantly higher IC_50_ values (20–19 µM in U-87 MG and 12–9 µM in U-138 MG), indicating lower cytotoxicity than ACT-containing combinations.

In normal MRC-5 fibroblasts, all formulations exhibited higher IC_50_ values as compared to the IC_50_ values of tumor cells, suggesting selective cytotoxicity toward cancer cells. ACT + NG nanoformulation treatment resulted in IC_50_ values of 10 µM and 8 µM at 24 and 48 h, respectively, whereas ACT+CBD achieved IC_50_ values of 12 µM and 9 µM. The control (DOTAP:POPC) formulation exhibited the least cytotoxicity, with IC_50_ values of 20 µM and 19 µM, for 24 and 48h, respectively.

These results suggest that the ACT + NG nanoformulation most effectively reduced glioma cell viability while exhibiting relatively low toxicity toward normal fibroblasts, highlighting its potential therapeutic value.

#### 3.3.2. Impact of Nanoformulations on Bax and Bcl-xL Protein Levels

The ability of cancer cells to evade apoptosis is a hallmark of cancer, while apoptosis is a promising target for all anticancer therapies [84]. Bcl-2 and Bax belong to the Bcl-2 family of proteins and are critical regulators of apoptosis, with Bcl-2 functioning as an anti-apoptotic protein and Bax as a pro-apoptotic protein. In cancer, overexpression of Bcl-2 and downregulation of Bax contribute to apoptosis resistance and uncontrolled cell proliferation [85].

**Figure 11:**
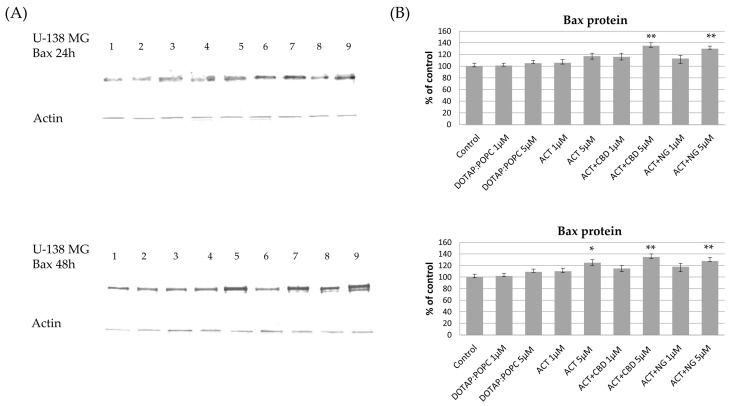
The impact of the nanoformulations on the level of Bax protein in U-138 MG cells. Panel (**A**) presents the representative immunoblots for the analysis of Bax protein. Panel (**B**) shows the densitometric analysis of Western blot results, which shows relative protein expression levels normalized to the control group. Data are presented as mean ± SEM from three independent experiments, expressed as fold change versus control. Statistical significance was assessed using Dunnett’s multiple comparisons test (* *p* < 0.05; ** *p* < 0.01).

**Figure 12:**
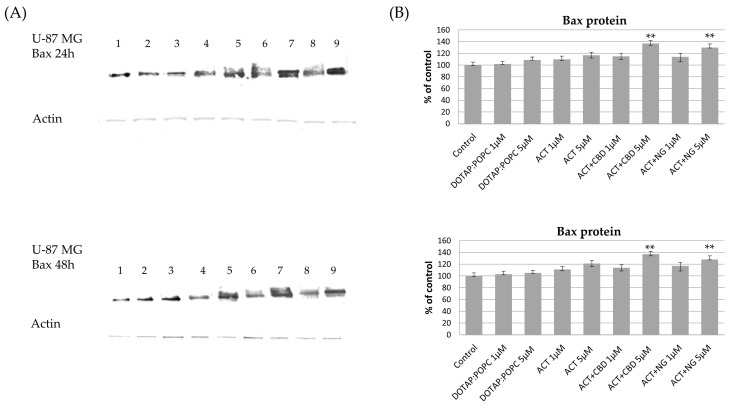
The impact of the nanoformulations on the level of Bax protein in U-87 MG cells. Panel (**A**) presents the representative immunoblots for the analysis of Bax protein. Panel (**B**) shows the sensitometric analysis of Western blot results, which shows relative protein expression levels normalized to the control group. Data are presented as mean ± SEM from three independent experiments, expressed as fold change versus control. Statistical significance was assessed using Dunnett’s multiple comparisons test (** *p* < 0.01).

**Figure 13:**
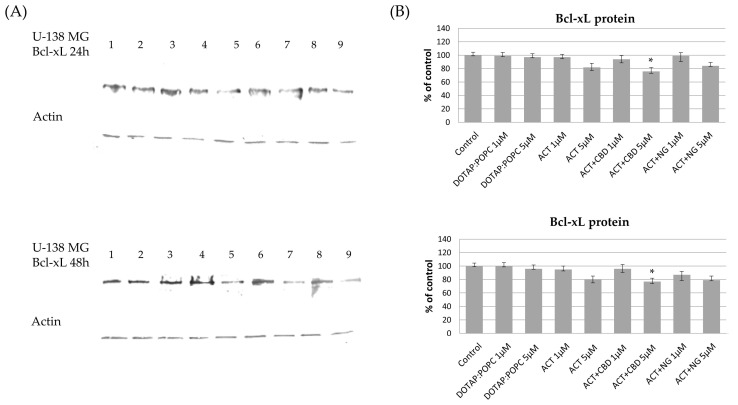
The impact of the nanoformulations on the level of Bcl-xL protein in U-138 MG cells. Panel (**A**) presents the representative immunoblots for the analysis of Bcl-xL protein. Panel (**B**) shows the densitometric analysis of Western blot results, which shows relative protein expression levels normalized to the control group. Data are presented as mean ± SEM from three independent experiments, expressed as fold change versus control. Statistical significance was assessed using Dunnett’s multiple comparisons test (* *p* < 0.05).

**Figure 14:**
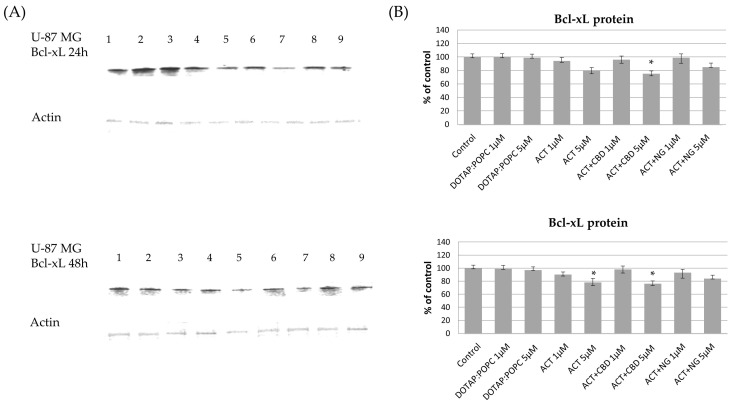
The impact of the nanoformulations on the level of Bcl-xL protein in U-87 MG cells. Panel (**A**) presents the representative immunoblots for the analysis of Bcl-xL protein. Panel (**B**) shows the densitometric analysis of Western blot results, which shows relative protein expression levels normalized to the control group. Data are presented as mean ± SEM from three independent experiments, expressed as fold change versus control. Statistical significance was assessed using Dunnett’s multiple comparisons test (* *p* < 0.05).

The effects of various nanoformulations on the expression levels of pro-apoptotic Bax and anti-apoptotic Bcl-xL proteins in U-87 MG and U-138 MG glioma cells were investigated using Western blot analysis at 24 and 48 h post-treatment. In both U-87 MG and U-138 MG cells, treatment with ACT (at 1 µM and 5 µM) and ACT-containing combinations with CBD or NG, led to a noticeable increase in Bax protein levels compared to the control nanoformulation. This dose-dependent effect became more pronounced after 48 h, indicating enhanced pro-apoptotic signaling. Treatment with control (DOTAP:POPC) nanoformulation alone had minimal impact on Bax protein expression (Figure 11 and Figure 12). For Bcl-xL, a decrease in protein levels was observed in both U-87 MG and U-138 MG cells following treatment with ACT-containing nanoformulations, particularly when combined with CBD or NG. The reduction was dose- and time-dependent, being more significant at higher doses and after 48 h of exposure. Treatment with control (DOTAP:POPC) formulation alone did not substantially alter Bcl-xL levels (Figure 13 and Figure 14).

Jia et al. found that ACT treatment reduced GBM tumor growth in a xenograft mouse model by increasing Bax and decreasing Bcl-2 expression, thereby promoting apoptosis in tumor cells [19]. Similarly, combining TMZ and ACT enhanced Bax and reduced Bcl-2 expression. This effect was more pronounced than with TMZ treatment alone, suggesting a synergistic impact on apoptosis in C6 cells [32].

Additionally, the study of Precilla et al. provided evidence that the co-delivery of TMZ and NG also exert pro-apoptotic effects. They showed that the combination of TMZ and NG suppressed C6 glioma cell growth in a dose-dependent manner, enhancing Bax expression and reducing Bcl-2 levels, which promoted apoptosis [86].

Patel et al. showed that CBD extracellular vesicles (5 mg/kg) promoted apoptosis by upregulating Bax and downregulating Bcl-2, and when combined with doxorubicin (500 nM), it further boosted Bax expression and lowered Bcl-2 in MDA-MB-231 cells [87].

In summary, the combination of ACT with CBD or NG in liposomes synergized by simultaneously upregulating pro-apoptotic Bax and downregulating anti-apoptotic Bcl-xL proteins, especially at higher concentrations and longer incubation times.

## 4. Conclusions

We demonstrated the potential for co-encapsulating ACT with other selected natural compounds, such as CBD and NG, in a cationic liposomal nanoformulation composed of two lipid types (DOTAP:POPC). The resulting nanoformulations were homogeneous and stable over 21 days, as demonstrated by a narrow particle size distribution, a low PDI index (<0.3), and a negative zeta potential. Furthermore, the production of liposomes smaller than 200 nm was confirmed, which is crucial for their potential clinical applications, including the treatment of GBM. The formulations exhibited a high encapsulation efficiency, exceeding 84%. In liposomes, additives such as CBD and NG influence the molecular dynamics of the materials, leading to reduced molecular mobility and enhanced interaction networks, modulating the particle size of the liposomal nanoformulation. By comparison, ACT alone maintains the most flexible dynamic behavior. All formulations exhibited higher IC_50_ values in MRC-5 fibroblasts than in glioma cells (U-87 MG and U-138 MG), indicating cancer-selective cytotoxicity. The ACT + NG nanoformulation showed the most potent cytotoxic effect in both glioma cell lines, with IC_50_ values decreasing to 6 µM in U-87 MG and 5 µM in U-138 MG after 48 h. The ACT + CBD combination was slightly less potent, with IC_50_ values of 7 µM in U-87 MG and 6 µM in U-138 MG after 48 h. The control (DOTAP:POPC) formulation showed higher IC_50_ values, indicating lower cytotoxicity than liposomes with ACT and NG/CBD. With CBD or NG, ACT-containing nanoformulations increased pro-apoptotic Bax. They decreased anti-apoptotic Bcl-xL levels in U-87 MG astrocytoma and U-138 MG GBM cells in a dose- and time-dependent manner. At the same time, the control formulation had minimal effect.

## Figures and Tables

**Table 1:** The Particle Size, PDI, and Zeta Potential of the liposomes (DOTAP:POPC) with ACT and the mixture of ACT and CBD or NG.

Time	Liposomes (DOTAP:POPC)	Particle Size (±SD) [nm]	PDI (±SD)	Zeta Potential (±SD) [mV]
T0	Control DOTAP:POPC	128.9 ± 5.0	0.21 ± 0.01	34.3 ± 1.7
	ACT	126.0 ± 7.7	0.17 ± 0.07	33.3 ± 3.6
	ACT + CBD	1273 ± 6.0	0.19 ± 0.05	37.3 ± 0.6
	ACT + NG	129.3 ± 25.1	0.17 ± 0.07	33.9 ± 3.3
T7	Control DOTAP:POPC	141.8 ± 40.3	0.21 ± 0.04	39.7 ± 0.4
	ACT	133.4 ± 16.3	0.21 ± 0.03	37.3 ± 2.8
	ACT + CBD	144.6 ± 9.6	0.24 ± 0.02	36.0 ± 2.5
	ACT + NG	142.2 ± 28.7	0.21 ± 0.07	34.6 ± 3.9
T14	Control DOTAP:POPC	154.3 ± 40.5	0.25 ± 0.03	38.6 ± 2.1
	ACT	190.6 ± 54.5	0.23 ± 0.03	38.7 ± 1.3
	ACT + CBD	153.4 ± 29.3	0.25 ± 0.04	37.3 ± 0.5
	ACT + NG	182.1 ± 59.3	0.23 ± 0.04	37.2 ± 3.8
T21	Control DOTAP:POPC	146.7 ± 39.1	0.23 ± 0.06	39.5 ± 4.1
	ACT	136.9 ± 6.4	0.21 ± 0.04	39.9 ± 2.1
	ACT + CBD	142.9 ± 18.5	0.22 ± 0.03	39.5 ± 3.2
	ACT + NG	147.8 ± 6.6	0.22 ± 0.08	34.1 ± 2.6

**Table 2:** Spin-lattice T_1_ and spin-spin T_2_ proton relaxation times measured in the laboratory frame.

Material	T_1_ (s)	$M_{0G}\;(\%)$	T_2G_ (ms)	$M_{0L}\;(\%)$	T_2L_ (ms)
ACT	2.5	58	184	42	300.0
ACT + CBD	2.6	77	232	23	7.6
ACT + NG	2.7	86	188	14	118.0
DOTAP:POPC	2.6	62	186	38	352.0

**Table 3:** Fitting parameters derived from applying Equation (6) to the experimental data.

Liposomal Systems	*K*	$\tau_{c}$ (ns)
ACT	1.22 ± 0.17	1.8
ACT + CBD	1.46 ± 0.27	2.8
ACT + NG	1.72 ± 0.21	3.6
DOTAP:POPC	1.61 ± 0.22	3.2

**Table 4:** The comparison of the viability of U-87, U-138MG, and MRC-5 cells—IC_50_ values (µM) following treatment with the tested nanoformulations.

Nanoformulations	U-87 MG 24 h	U-87 MG 48 h	U-138 MG 24 h	U-138 MG 48 h	MRC-524 h	MRC-548 h
DOTAP:POPC	18	16	17	15	20	19
ACT + CBD	11	7	10	6	12	9
ACT + NG	9	6	8	5	10	8

## Data Availability

The original contributions presented in this study are included in the article. Further inquiries can be directed to the corresponding authors.

## Abbreviations

The following abbreviations are used in this manuscript:

ACT	Acteoside
BBB	Blood-brain barrier
CBD	Cannabidiol
CUR	Curcumin
CNS	Central nervous system
DDS	Drug delivery system
DLS	Dynamic light scattering
DOTAP	1,2-dioleoyl-3-trimethylammonium-propane
ELS	Electrophoretic light scattering
GBM	Glioblastoma
HRP	Horseradish peroxidase
LUVs	Large unilamellar vesicles
LDDS	Lipid based drug delivery systems
MTT	3-(4,5-dimethylthiazol-2-yl)-2,5-diphenyltetrazolium bromide
MLVs	Multilamellar vesicles
NMR	Nuclear magnetic resonance
NG	Naringenin
ORI	Orientin
PBS	Phosphate-buffered saline
PDI	Polydispersity index
POPC	1-Palmitoyl-2-oleoyl-glycero-3-phosphocholine
RQ	Relative quantification
SUVs	Small unilamellar vesicles
TMZ	Temozolomide

## References

[1] Yang Y.-C., Zhu Y., Sun S.-J., Zhao C.-J., Bai Y., Wang J., Ma L.-T. (2023). ROS Regulation in Gliomas: Implications for Treatment Strategies. Front. Immunol..

[2] Antonelli M., Poliani P.L. (2022). Adult Type Diffuse Gliomas in the New 2021 WHO Classification. Pathologica.

[3] Paw I., Carpenter R.C., Watabe K., Debinski W., Lo H.-W. (2015). Mechanisms Regulating Glioma Invasion. Cancer Lett..

[4] Rodriguez B., Brown C.S., Colan J.A., Zhang J.Y., Huq S., Rivera D., Young T., Williams T., Subramaniam V., Hadjipanayis C. (2025). Fluorescence-Guided Surgery for Gliomas: Past, Present, and Future. Cancers.

[5] Veviorskiy A., Mkrtchyan G.V., Osipov A.N., Izumchenko E., Ozerov I.V., Aliper A., Zhavoronkov A., Scheibye-Knudsen M. (2025). Variability in Radiotherapy Outcomes across Cancer Types: A Comparative Study of Glioblastoma Multiforme and Low-Grade Gliomas. Aging.

[6] Rominiyi O., Collis S.J. (2022). DDRugging Glioblastoma: Understanding and Targeting the DNA Damage Response to Improve Future Therapies. Mol. Oncol..

[7] Koosha F., Ahmadikamalabadi M., Mohammadi M. (2024). Review of Recent Improvements in Carbon Ion Radiation Therapy in the Treatment of Glioblastoma. Adv. Radiat. Oncol..

[8] Das S., Marsden P.A. (2013). Angiogenesis in Glioblastoma. N. Engl. J. Med..

[9] Schmassmann P., Roux J., Dettling S., Hogan S., Shekarian T., Martins T.A., Ritz M.-F., Herter S., Bacac M., Hutter G. (2023). Single-Cell Characterization of Human GBM Reveals Regional Differences in Tumor-Infiltrating Leukocyte Activation. eLife.

[10] Yasinjan F., Xing Y., Geng H., Guo R., Yang L., Liu Z., Wang H. (2023). Immunotherapy: A Promising Approach for Glioma Treatment. Front. Immunol..

[11] Chan P., Rich J.N., Kay S.A. (2023). Watching the Clock in Glioblastoma. Neuro-Oncology.

[12] Kanderi T., Munakomi S., Gupta V. (2024). Glioblastoma Multiforme. StatPearls.

[13] Dipasquale A., Franceschi E., Giordano L., Maccari M., Barigazzi C., Di Nunno V., Losurdo A., Persico P., Di Muzio A., Navarria P. (2024). Dissecting the Prognostic Signature of Patients with Astrocytoma Isocitrate Dehydrogenase-Mutant Grade 4: A Large Multicenter, Retrospective Study. ESMO Open.

[14] Majchrzak-Celińska A., Studzińska-Sroka E. (2024). New Avenues and Major Achievements in Phytocompounds Research for Glioblastoma Therapy. Molecules.

[15] Zhao Y., Wang S., Pan J., Ma K. (2023). Verbascoside: A Neuroprotective Phenylethanoid Glycosides with Anti-Depressive Properties. Phytomedicine.

[16] Gao L., Peng X.-M., Huo S.-X., Liu X.-M., Yan M. (2015). Memory Enhancement of Acteoside (Verbascoside) in a Senescent Mice Model Induced by a Combination of d-Gal and AlCl3. Phytother. Res..

[17] Khan R.A., Hossain R., Roy P., Jain D., Mohammad Saikat A.S., Roy Shuvo A.P., Akram M., Elbossaty W.F., Khan I.N., Painuli S. (2022). Anticancer Effects of Acteoside: Mechanistic Insights and Therapeutic Status. Eur. J. Pharmacol..

[18] Xiao Y., Ren Q., Wu L. (2022). The Pharmacokinetic Property and Pharmacological Activity of Acteoside: A Review. Biomed. Pharmacother..

[19] Jia W.-Q., Wang Z.-T., Zou M.-M., Lin J.-H., Li Y.-H., Zhang L., Xu R.-X. (2018). Verbascoside Inhibits Glioblastoma Cell Proliferation, Migration and Invasion While Promoting Apoptosis Through Upregulation of Protein Tyrosine Phosphatase SHP-1 and Inhibition of STAT3 Phosphorylation. Cell. Physiol. Biochem..

[20] Jia W.-Q., Zhu J.-W., Yang C.-Y., Ma J., Pu T.-Y., Han G.-Q., Zou M.-M., Xu R.-X. (2020). Verbascoside Inhibits Progression of Glioblastoma Cells by Promoting Let-7g-5p and down-Regulating HMGA2 via Wnt/Beta-Catenin Signalling Blockade. J. Cell. Mol. Med..

[21] Heider C.G., Itenberg S.A., Rao J., Ma H., Wu X. (2022). Mechanisms of Cannabidiol (CBD) in Cancer Treatment: A Review. Biology.

[22] Mokoena D., George B.P., Abrahamse H. (2024). Cannabidiol Combination Enhances Photodynamic Therapy Effects on MCF-7 Breast Cancer Cells. Cells.

[23] Rybarczyk A., Majchrzak-Celińska A., Krajka-Kuźniak V. (2023). Targeting Nrf2 Signaling Pathway in Cancer Prevention and Treatment: The Role of Cannabis Compounds. Antioxidants.

[24] Solinas M., Massi P., Cinquina V., Valenti M., Bolognini D., Gariboldi M., Monti E., Rubino T., Parolaro D. (2013). Cannabidiol, a Non-Psychoactive Cannabinoid Compound, Inhibits Proliferation and Invasion in U87-MG and T98G Glioma Cells through a Multitarget Effect. PLoS ONE.

[25] de Santana M.R., Argolo D.S., Lima I.S., dos Santos C.C., Victor M.M., Ramos G.d.S., do Nascimento R.P., Ulrich H., Costa S.L. (2025). Naringenin Exhibits Antiglioma Activity Related to Aryl Hydrocarbon Receptor Activity and IL-6, CCL2, and TNF-α Expression. Brain Sci..

[26] Atoki A.V., Aja P.M., Shinkafi T.S., Ondari E.N., Awuchi C.G. (2024). Naringenin: Its Chemistry and Roles in Neuroprotection. Nutr. Neurosci..

[27] Zhu Y., Guo X., Li S., Wu Y., Zhu F., Qin C., Zhang Q., Yang Y. (2024). Naringenin Ameliorates Amyloid-β Pathology and Neuroinflammation in Alzheimer’s Disease. Commun. Biol..

[28] Uçar K., Göktaş Z. (2023). Biological Activities of Naringenin: A Narrative Review Based on in Vitro and in Vivo Studies. Nutr. Res..

[29] Dhiman A., Shah Y., Rana D., Garkhal K. (2025). Comprehensive Review on Glioblastoma: Nanotechnology, Immunotherapy and Combined Therapeutic Approaches. RSC Pharm..

[30] Hong J.P., Choi R.J., Shim J.-K., Kim K., Kim R.N., Cho H., Kim S.J., Kim S., Kim N.H., Park H.H. (2025). Synergistic Combination of Perphenazine and Temozolomide Suppresses Patient-Derived Glioblastoma Tumorspheres. Neuro-Oncology.

[31] Dhungel L., Rowsey M.E., Harris C., Raucher D. (2024). Synergistic Effects of Temozolomide and Doxorubicin in the Treatment of Glioblastoma Multiforme: Enhancing Efficacy through Combination Therapy. Molecules.

[32] Hwang T.W., Kim D.H., Kim D.B., Jang T.W., Kim G.-H., Moon M., Yoon K.A., Choi D.E., Park J.H., Kim J.-J. (2019). Synergistic Anticancer Effect of Acteoside and Temozolomide-Based Glioblastoma Chemotherapy. Int. J. Mol. Med..

[33] Soroceanu L., Singer E., Dighe P., Sidorov M., Limbad C., Rodriquez-Brotons A., Rix P., Woo R.W.L., Dickinson L., Desprez P.-Y. (2022). Cannabidiol Inhibits RAD51 and Sensitizes Glioblastoma to Temozolomide in Multiple Orthotopic Tumor Models. Neurooncol Adv..

[34] Gautam M., Gabrani R. (2022). Combinatorial Effect of Temozolomide and Naringenin in Human Glioblastoma Multiforme Cell Lines. Nutr. Cancer.

[35] Grzegorzewski J., Michalak M., Wołoszczuk M., Bulicz M., Majchrzak-Celińska A. (2025). Nanotherapy of Glioblastoma—Where Hope Grows. Int. J. Mol. Sci..

[36] Yaroslavov A.A., Efimova A.A., Krasnikov E.A., Trosheva K.S., Popov A.S., Melik-Nubarov N.S., Krivtsov G.G. (2021). Chitosan-Based Multi-Liposomal Complexes: Synthesis, Biodegradability and Cytotoxicity. Int. J. Biol. Macromol..

[37] Zhang Y., Liu B., Wu H., Li B., Xu J., Duan L., Jiang C., Zhao X., Yuan Y., Zhang G. (2014). Anti-Tumor Activity of Verbascoside Loaded Gold Nanoparticles. J. Biomed. Nanotechnol..

[38] Ma Z., Zhao X., Jiang C., Yu J., Wu J., Zeng X. (2016). Gold Nanoshells with Verbascoside Induce the Apoptosis of Drug-Resistant Leukemia Cells Through Caspases Pathway and Inhibit Tumor Growth. J. Nanosci. Nanotechnol..

[39] Zhao X.-H., Yue H.-L., Li P., Zeng X., Zhang G. (2013). Evaluation of the Antitumor Activity by CdTe QDs with Verbascoside. Nano.

[40] Alcantara K.P., Malabanan J.W.T., Nalinratana N., Thitikornpong W., Rojsitthisak P., Rojsitthisak P. (2024). Cannabidiol-Loaded Solid Lipid Nanoparticles Ameliorate the Inhibition of Proinflammatory Cytokines and Free Radicals in an In Vitro Inflammation-Induced Cell Model. Int. J. Mol. Sci..

[41] Qayum S., Schmitt R.R., Machhar J.S., Garg S., Bass C., Muthaiah V.P.K., Ignatowski T.A., Mahajan S.D. (2024). Biodegradable Cannabidiol: A Potential Nanotherapeutic for Neuropathic Pain. NeuroImmune Pharmacol. Ther..

[42] Mahanta A.K., Chaulagain B., Trivedi R., Singh J. (2024). Mannose-Functionalized Chitosan-Coated PLGA Nanoparticles for Brain-Targeted Codelivery of CBD and BDNF for the Treatment of Alzheimer’s Disease. ACS Chem. Neurosci..

[43] Taha I.E., ElSohly M.A., Radwan M.M., Elkanayati R.M., Wanas A., Joshi P.H., Ashour E.A. (2025). Enhancement of Cannabidiol Oral Bioavailability through the Development of Nanostructured Lipid Carriers: In Vitro and in Vivo Evaluation Studies. Drug Deliv. Transl. Res..

[44] Nie Y., Kong Y., Peng J., Sun J., Fan B. (2024). Enhanced Oral Bioavailability of Cannabidiol by Flexible Zein Nanoparticles: In Vitro and Pharmacokinetic Studies. Front. Nutr..

[45] Maity S., Chakraborti A.S. (2020). Formulation, Physico-Chemical Characterization and Antidiabetic Potential of Naringenin-Loaded Poly D, L Lactide-Co-Glycolide (N-PLGA) Nanoparticles. Eur. Polym. J..

[46] Gera S., Talluri S., Rangaraj N., Sampathi S. (2017). Formulation and Evaluation of Naringenin Nanosuspensions for Bioavailability Enhancement. AAPS PharmSciTech.

[47] Nouri Z., Sajadimajd S., Hoseinzadeh L., Bahrami G., Arkan E., Moradi S., Abdi F., Farzaei M.H. (2022). Neuroprotective Effect of Naringenin-Loaded Solid Lipid Nanoparticles against Streptozocin-Induced Neurotoxicity through Autophagy Blockage. J. Food Biochem..

[48] Pour P.M., Nouri Z., Ghasemi D., Sajadimajd S., Farzaei M.H. (2024). Cytotoxic Impact of Naringenin-Loaded Solid Lipid Nanoparticles on RIN5F Pancreatic β Cells via Autophagy Blockage. Recent Adv. Drug Deliv. Formul..

[49] Mobaleghol Eslam H., Hataminia F., Esmaeili F., Salami S.A., Ghanbari H., Amani A. (2024). Preparation of a Nanoemulsion Containing Active Ingredients of Cannabis Extract and Its Application for Glioblastoma: In Vitro and in Vivo Studies. BMC Pharmacol. Toxicol..

[50] Juhairiyah F., de Lange E.C.M. (2021). Understanding Drug Delivery to the Brain Using Liposome-Based Strategies: Studies That Provide Mechanistic Insights Are Essential. AAPS J..

[51] Joshi S., Singh-Moon R.P., Ellis J.A., Chaudhuri D.B., Wang M., Reif R., Bruce J.N., Bigio I.J., Straubinger R.M. (2015). Cerebral Hypoperfusion-Assisted Intra-Arterial Deposition of Liposomes in Normal and Glioma-Bearing Rats. Neurosurgery.

[52] Piwowarczyk L., Mlynarczyk D.T., Krajka-Kuźniak V., Majchrzak-Celińska A., Budzianowska A., Tomczak S., Budzianowski J., Woźniak-Braszak A., Pietrzyk R., Baranowski M. (2022). Natural Compounds in Liposomal Nanoformulations of Potential Clinical Application in Glioblastoma. Cancers.

[53] Piwowarczyk L., Kucinska M., Tomczak S., Mlynarczyk D.T., Piskorz J., Goslinski T., Murias M., Jelinska A. (2022). Liposomal Nanoformulation as a Carrier for Curcumin and pEGCG—Study on Stability and Anticancer Potential. Nanomaterials.

[54] ICH Q2(R2) Guideline on Validation of Analytical Procedures–Step 5. https://www.ema.europa.eu/en/ich-q2r2-validation-analytical-procedures-scientific-guideline.

[55] Hudiyanti D., Al Khafiz M.F., Anam K., Siahaan P., Christa S.M. (2022). In Vitro Evaluation of Curcumin Encapsulation in Gum Arabic Dispersions under Different Environments. Molecules.

[56] Ma J., Guo C., Tang Y., Xiang J., Chen S., Wang J., Liu H. (2007). Micellization in Aqueous Solution of an Ethylene Oxide–Propylene Oxide Triblock Copolymer, Investigated with 1H NMR Spectroscopy, Pulsed-Field Gradient NMR, and NMR Relaxation. J. Colloid Interface Sci..

[57] Inoue T. (2009). Micelle Formation of Polyoxyethylene-Type Nonionic Surfactants in bmimBF4 Studied by 1H NMR and Dynamic Light-Scattering. J. Colloid Interface Sci..

[58] Leal C., Rögnvaldsson S., Fossheim S., Nilssen E.A., Topgaard D. (2008). Dynamic and Structural Aspects of PEGylated Liposomes Monitored by NMR. J. Colloid Interface Sci..

[59] Niklasson M., Otten R., Ahlner A., Andresen C., Schlagnitweit J., Petzold K., Lundström P. (2017). Comprehensive Analysis of NMR Data Using Advanced Line Shape Fitting. J. Biomol. NMR.

[60] Slichter C.P. (1978). Principles of Magnetic Resonance.

[61] Faid K., Fox R.F. (1986). Stochastic Theory of Line Shape and Relaxation. Phys. Rev. A.

[62] Weiger M., Pruessmann K.P. (2019). Short-T2 MRI: Principles and Recent Advances. Prog. Nucl. Magn. Reson. Spectrosc..

[63] Lasic D.D. (1991). Magnetic Resonance Methods in The Studies of Liposomes. Bull. Magn. Reason..

[64] Baranowski M., Woźniak-Braszak A., Jurga K. (2011). High Homogeneity B1 30.2 MHz Nuclear Magnetic Resonance Probe for off-Resonance Relaxation Times Measurements. J. Magn. Reson..

[65] Desvaux H., Goldman M. (1994). A New NMR Method for Measuring the Rotational Correlation Time of Molecules in the Liquid State. Mol. Phys..

[66] Gilani I.A., Sepponen R. (2016). Quantitative Rotating Frame Relaxometry Methods in MRI. NMR Biomed..

[67] Kimmich R., Fatkullin N. (2017). Self-Diffusion Studies by Intra- and Inter-Molecular Spin-Lattice Relaxometry Using Field-Cycling: Liquids, Plastic Crystals, Porous Media, and Polymer Segments. Prog. Nucl. Magn. Reson. Spectrosc..

[68] Jurga K., Fojud Z., Woźniak-Braszak A. (2004). NMR Strong Off-Resonance Irradiation without Sample Overheating. Solid State Nucl. Magn. Reson..

[69] Makrocka-Rydzyk M., Woźniak-Braszak A., Jurga K., Jurga S. (2015). Local Motions in Poly(Ethylene-*Co*-Norbornene) Studied by 1H NMR Relaxometry. Solid State Nucl. Magn. Reson..

[70] Danaei M., Dehghankhold M., Ataei S., Hasanzadeh Davarani F., Javanmard R., Dokhani A., Khorasani S., Mozafari M.R. (2018). Impact of Particle Size and Polydispersity Index on the Clinical Applications of Lipidic Nanocarrier Systems. Pharmaceutics.

[71] Wu Y.-T., Lin L.-C., Sung J.-S., Tsai T.-H. (2006). Determination of Acteoside in Cistanche Deserticola and Boschniakia Rossica and Its Pharmacokinetics in Freely-Moving Rats Using LC-MS/MS. J. Chromatogr. B Anal. Technol. Biomed. Life Sci..

[72] Isacchi B., Bergonzi M.C., Iacopi R., Ghelardini C., Galeotti N., Bilia A.R. (2016). Liposomal Formulation to Increase Stability and Prolong Antineuropathic Activity of Verbascoside. Planta Medica.

[73] Sitovs A., Logviss K., Lauberte L., Mohylyuk V. (2024). Oral Delivery of Cannabidiol: Revealing the Formulation and Absorption Challenges. J. Drug Deliv. Sci. Technol..

[74] Zgair A., Wong J.C., Lee J.B., Mistry J., Sivak O., Wasan K.M., Hennig I.M., Barrett D.A., Constantinescu C.S., Fischer P.M. (2016). Dietary Fats and Pharmaceutical Lipid Excipients Increase Systemic Exposure to Orally Administered Cannabis and Cannabis-Based Medicines. Am. J. Transl. Res..

[75] Millar S.A., Stone N.L., Yates A.S., O’Sullivan S.E. (2018). A Systematic Review on the Pharmacokinetics of Cannabidiol in Humans. Front. Pharmacol..

[76] Tsai M.-J., Huang Y.-B., Fang J.-W., Fu Y.-S., Wu P.-C. (2015). Preparation and Characterization of Naringenin-Loaded Elastic Liposomes for Topical Application. PLoS ONE.

[77] Wang Y., Wang S., Firempong C.K., Zhang H., Wang M., Zhang Y., Zhu Y., Yu J., Xu X. (2017). Enhanced Solubility and Bioavailability of Naringenin via Liposomal Nanoformulation: Preparation and In Vitro and In Vivo Evaluations. AAPS PharmSciTech.

[78] Sahoo L., Tripathy N.S., Dilnawaz F. (2024). Naringenin Nanoformulations for Neurodegenerative Diseases. Curr. Pharm. Biotechnol..

[79] Nikolova M.P., Kumar E.M., Chavali M.S. (2022). Updates on Responsive Drug Delivery Based on Liposome Vehicles for Cancer Treatment. Pharmaceutics.

[80] Smith M.C., Crist R.M., Clogston J.D., McNeil S.E. (2017). Zeta Potential: A Case Study of Cationic, Anionic, and Neutral Liposomes. Anal. Bioanal. Chem..

[81] Fan Y., Marioli M., Zhang K. (2021). Analytical Characterization of Liposomes and Other Lipid Nanoparticles for Drug Delivery. J. Pharm. Biomed. Anal..

[82] Woźniak-Braszak A., Jurga K., Baranowski M. (2016). The Lipari-Szabo Model-Free Analysis as a Method for Study of Molecular Motion in Solid State Heteronuclear Systems Using NMR Off-Resonance. Appl. Magn. Reson..

[83] Singh K., Bhushan B., Chanchal D.K., Sharma S.K., Rani K., Yadav M.K., Porwal P., Kumar S., Sharma A., Virmani T. (2023). Emerging Therapeutic Potential of Cannabidiol (CBD) in Neurological Disorders: A Comprehensive Review. Behav. Neurol..

[84] Dakkak B.E., Taneera J., El-Huneidi W., Abu-Gharbieh E., Hamoudi R., Semreen M.H., Soares N.C., Abu-Rish E.Y., Alkawareek M.Y., Alkilany A.M. (2024). Unlocking the Therapeutic Potential of BCL-2 Associated Protein Family: Exploring BCL-2 Inhibitors in Cancer Therapy. Biomol. Ther..

[85] Huang Y.-K., Chang K.-C., Li C.-Y., Lieu A.-S., Lin C.-L. (2023). AKR1B1 Represses Glioma Cell Proliferation through P38 MAPK-Mediated Bcl-2/BAX/Caspase-3 Apoptotic Signaling Pathways. Curr. Issues Mol. Biol..

[86] Daisy P.S., Shreyas S.K., Sathish R., Anitha T.S. (2021). Synergistic Apoptotic Effect of Naringenin on Enhancing the Anti-Glioma Efficacy of Temozolomide in an in Vitro Experimental Model. Res. J. Biotechnol..

[87] Patel N., Kommineni N., Surapaneni S.K., Kalvala A., Yaun X., Gebeyehu A., Arthur P., Duke L.C., York S.B., Bagde A. (2021). Cannabidiol Loaded Extracellular Vesicles Sensitize Triple-Negative Breast Cancer to Doxorubicin in Both In-Vitro and In Vivo Models. Int. J. Pharm..

